# Host Gene Polymorphisms in Relation to Helicobacter Pylori Infection and Associated Diseases in a Population Based Cohort

**DOI:** 10.4021/gr578w

**Published:** 2014-01-15

**Authors:** Anna Ryberg, Fredrik Petersson, Stefan Redeen, Olle Eriksson, Kurt Borch

**Affiliations:** a Department of Clinical Microbiology, Centre for Diagnostics, County Council of Ostergotland, Sweden; b Department of Clinical and Experimental Medicine, Faculty of Health Sciences, Linkoping University, Sweden; c Department of Pathology, National University Health System Singapore. Previously Department of Pathology, Karolinska University Hospital, Stockholm, Sweden; d Department of Computer and Information Science, Faculty of Arts and Sciences, Linkoping University, Sweden

**Keywords:** Atrophy, Gastritis, Peptic ulcer, Pyrosequencing, Stomach

## Abstract

**Background:**

This prospective population based cohort study explores possible associations between host gene polymorphisms, blood group and life style factors on the one hand, and *Helicobacter pylori* infection, peptic ulcer, and the grade of inflammation, atrophy and intestinal metaplasia of the gastric mucosa, on the other hand.

**Methods:**

The study population (472 volunteers) has previously undergone screening with gastroduodenoscopy, biopsy and blood sampling. The host gene polymorphisms of IL1B-31C/T, IFNGR1-56T/C, the IL1RN VNTR in exon 2 and the HLA-DRB1 gene alleles were analyzed using PCR and pyrosequencing.

**Results:**

*H. pylori* infection was negatively related to HLA DRB1*03 (odds ratio (OR) 95% CI: 0.388 - 0.989) and was more frequent in individuals with blood group O than A (OR 95% CI: 1.121 - 2.677). There was a lower risk of moderate to severe inflammation in the antrum among individuals with IL1B-31 TC compared to CC carriers (OR 95% CI: 0.094 - 0.733). The IL1RN*L2 genotype was associated with higher risk of IM in the antrum than the *LL genotype (OR 95% CI: 1.570 - 15.878). There was a negative relation between the HLA DRB1 alleles *04 (OR 95% CI: 0.234 - 0.831) and *08 (OR 95% CI: 0.013 - 0.915), and IM in the antrum.

**Conclusion:**

The IL1RN VNTR and the IL1β-31 alleles seem to be associated with intestinal metaplasia of the corpus mucosa and the grade of inflammation of the antrum, respectively. However, no unambiguous correlations could be identified between the host polymorphisms and the occurrence of *H. pylori* infection, peptic ulcer, and the grade of inflammation, atrophy and IM of the gastric mucosa.

## Introduction

*Helicobacter pylori* (*H. pylori*) infection is associated with gastroduodenal diseases, such as peptic ulcer (PU), the premalignant condition atrophic gastritis (AG), gastric carcinoma (GC) and gastric mucosa associated lymphoid tissue (MALT) lymphoma [1]. Infection with these Gram-negative, microaerophilic bacteria is often life long, but exactly how *H. pylori* eludes the immune defence system is still not clear. Previous studies indicate that a combination of host gene polymorphisms, *H. pylori* virulence genes and environmental factors determines the outcome of the infection [2, 3].

Several host gene variations have been related to *H. pylori* infection and the development of associated gastroduodenal diseases. Interleukin-1β (IL1β) and interleukin-1 receptor antagonist (IL1RN) gene polymorphisms have been found to be associated with increased risk of AG, GC and duodenal ulcer (DU) [4, 5], but contradictory results have been reported [6, 7].

A recent meta-analysis [7] concluded that the IL1 receptor antagonist gene contains a variable number of tandem repeats (VNTR), where a short variant, containing two 86bp-repeats, is associated with GC, specifically in non-Asian populations. However, the authors could not find an overall correlation between cancer and polymorphisms in the promoter region of the very potent gastric acid secretion inhibitor IL1β, except the finding of a reduced risk of cancer in IL1β-31C carriers in Asian populations. An earlier meta-analysis [8] concluded that there is an increased risk of GC associated with IL1B-511T and IL1RN*2 alleles in Caucasians, but not in Asians, and that there is a trend towards an association between IL1B-31C and GC in Caucasians.

IFNGR1 is the ligand-binding subunit of the interferon gamma receptor dimer. By genome wide linkage analysis, Thye et al (2003) [9] found increased anti-*H. pylori* serum immunoglobulin G levels among Senegalese siblings, who were homozygous or heterozygous carriers of the IFNGR1-56T variant. Since then, several studies have indicated that there is a more general association between -56C/T SNP and human pathology related to both bacteria and viruses, the IFNGR1-56CC genotype being associated with protection from TB [10] and -56C with spontaneous clearance of hepatitis B virus [11]. Zhou et al [11] also showed that the IFNGR-56C variant is associated with higher transcription level than -56T.

The human leukocyte antigen gene DRB1 encodes the beta subunit of the HLA class II complex, presenting peptides on the surface of antigen presenting cells [12]. In Japan, the HLA gene DRB1*0405 allele was associated with duodenal ulcer [13]. The DRB1*04051 has been shown to be associated with gastric adenocarcinoma independent of *H. pylori* infection [14], and the DRB1*1501 was negatively related to gastric ulcer, duodenal ulcer and *H. pylori*-associated gastritis when compared to non-infected healthy individuals [13]. The DRB1*1601 allele was found to be associated with increased risk of the diffuse type of stomach cancer in a Swedish population, especially in *H. pylori* negative individuals [15].

The aim of this study was to explore whether there are any associations between the occurrence of IL-1B-31T/C, IL1RN VNTR alleles, IFNGR1-56C/T, HLA DRB1 alleles, together with blood group and life style factors on the one hand, and the occurrence of *H. pylori* infection, peptic ulcer and the grade of inflammation, atrophy and intestinal metaplasia (IM) of the gastric mucosa, on the other hand, in a prospective population based cohort in Sweden.

## Methods

### Study population and material

The study was conducted in accordance with the Helsinki declaration and was approved by the Regional Ethics Committee of Southeast of Sweden. Informed written consent was obtained from all participants. The study population is a cohort of 472 individuals from a larger (n = 506 volunteers) population study [16, 17]. The study includes all individuals that underwent screening with gastroduodenoscopy, biopsy and blood sampling [16] (fasting state), and from whom DNA of sufficient quality for genotyping analysis could be isolated. There were 218 females and 254 males included in the study. Histologic examination of biopsies was performed as previously described [16]. Gastritis was classified according to the Sydney system [16, 18, 19]. The prevalence of chronic gastritis in the corpus was 9.5% (48.9% males) and moderate to severe chronic gastritis was observed in 4.0% of the volunteers (52.6% males). All ulcers diagnosed at gastroduodenoscopy were biopsied. No malignant ulcers were encountered. According to gastroduodenoscopy and histologic examination of biopsies, 8.7% of the study population had benign ulcer (58.5% males) and 3.8% had ulcer exclusively located in the duodenum (72.2% males).

Blood samples were stored at -80 °C until analysis. *H. pylori* status was classified as positive when at least two of the following occurred: *H. pylori* identified by light microscopic examination (Giemsa-stained sections), positive result of urease test on fresh biopsy specimen, or elevated level of *H. pylori* IgG antibodies in serum [16], 187 individuals fulfilled the criteria for positive *H. pylori* status (54% males). Serum pepsinogen I (PGI) and II (PGII) concentrations were analyzed using sandwich enzyme immunoassay (ELISA) as previously described [17]. The reference interval is 2.0 - 20.0 for the ratio PGI/PGII and values lower than 3.0 are considered indicative of significant atrophy of the gastric corpus mucosa.

H, K-ATPase IgG antibodies were analyzed by ELISA as previously described [20] and results are given as relative optical density (OD; upper normal limit 15%). *H. pylori* IgG antibodies against surface antigens were analyzed with the same method (upper normal limit of OD 5% [17]).

### Isolation of DNA and whole genome amplification

DNA was extracted from whole blood at the Department of Forensic Genetics of the Swedish National Board of Forensic Medicine (Linkoping, Sweden), using the M48 BioRobot (Qiagen, Hilden, Germany). In cases from whom no whole blood was available for various reasons, DNA was extracted from plasma or serum using either the QIAamp DNA mini isolation kit (Qiagen, Hilden, Germany) following the blood and body fluid spin protocol, or MagAttract DNA mini M48 kit following the cultured cell protocol. In short, 200 - 400 µL plasma/serum was used and 5 µg dA/dT DNA carrier probe (GE Healthcare, Uppsala, Sweden) was included in the manual extraction. For automatic extraction using the M48 BioRobot (Qiagen, Hilden, Germany) 400 µL plasma/serum was centrifuged at 3,000 *g* for 30 min at 4 °C, the pellet resuspended in 190 µL buffer G2 and 10 µL proteinase K was added, the samples were incubated at 37 °C for 0.5 - 1 h before extraction and 1 µL DNA was used for whole genome amplification according to the GenomiPhi DNA amplification kit (GE Healthcare, Uppsala, Sweden). MDA-DNA concentrations were determined using an ND-1000 spectrophotometer (Nanodrop Technologies, Wilmington, DE, USA).

### PCR analysis

The presence of amplified DNA was verified using human 18S primers as previously described [21]. Amplification and pyrosequencing analysis of IL1B SNPs rs1143627 (-31C/T), and IFNGR1 SNP rs2234711 (-56T/C), and amplification and gel electrophoresis analysis of IL1RN VNTR in exon 2 were done as previously described [21]. The HLA-DRB1 gene was analyzed at the Department of Forensic Genetics of the Swedish National Board of Forensic Medicine (Linkoping, Sweden), following the recommendation for the HLA-DR low kit (Olerup, Norway).

### Statistical analysis

The continuous dependent variable PGI/PGII was analyzed using general linear model (GLM) and Minitab (v. 15). All other dependent variables were dichotomous, and were analyzed using forward stepwise (Wald method) binary logistic regression (BLR) and the SPSS statistical analysis program (v. 17). The Hosmer and Lemeshow goodness of fit test was checked at the last step for verification of each analysis. A P-value of < 0.05 was considered significant.

The independent variables used in the GLM and BLR analyses were: relative OD of H,K-ATPase antibodies, *H. pylori* infection (this variable was excluded when used as dependent), gender, BMI, age, use of non-steroidal anti-inflammatory drugs (NSAID; including low-dose aspirin) on a weekly basis, smoking (yes/no), alcohol consumption (yes/no on a weekly basis [16]), ABO blood group (A, B, 0, and AB), Rhesus blood group (+ and -), IL1B genotypes, IFNGR1 genotypes, IL1RN genotypes, and carriers of allele HLA-DRB1*01, DRB1*03, DRB1*04, DRB1*07, DRB1*08, DRB1*11, DRB1*13 and DRB1*15. The chosen HLA-DRB1 alleles were the most frequent (at least 30 alleles in the study counting both homozygote and heterozygote cases (supplementary 1, www.gastrores.org).

## Results

### DNA isolation and quality control

The MDA reaction yielded approximately 750 ng/µL MDA-DNA in each amplification reaction, and 10 samples were excluded from further analysis due to low quality of DNA, even after repetitive DNA isolation attempts. All included samples (472) generated 18S and cytokine PCR products of expected sizes.

### Genotype distribution

The genotype frequency distribution of all analyzed cytokine polymorphisms in this study are presented in supplementary 2 (www.gastrores.org). The distribution of IL1B-31 genotype TT/TC/CC was 42.8/42.2/15.0%. For IFNGR1-56 the distribution of genotype TT/TC/CC was 44.1/42.6/13.3%. The IL1RN 86bp VNTR genotypes were classified into allele 2 (2 repeats) or L for three or more repeats [4], and the distribution of genotypes LL/L2/22 was 50.0/39.2/10.8%. The frequency of HLA-DRB1 allele types is shown in supplementary 1 (www.gastrores.org).

### Statistical analysis

A summary of all analyses in which the regression coefficient was significantly different from zero is shown in Table 1. The results are grouped according to the dependent variable analyzed; *H. pylori* status, scores for chronic inflammation of the corpus and antrum mucosa, ulcer overall (duodenal or gastric), duodenal ulcer, any degree of atrophy of the corpus mucosa, moderate to severe atrophy of the corpus mucosa, PGI/PGII as surrogate marker for atrophy of the corpus mucosa, and IM of the corpus and antrum mucosa. The details of each variable analysed are presented below in five parts: *H. pylori* infection, inflammation, ulcer, atrophy and IM.

**Table 1:** Summary of Statistically Significant Findings in the Study

Independent variables	Dependent variables												
	*H. pylori* infection	Infl. antrum		Infl. corpus		Ulcer		Atrophy of corpus			IM antrum		IM in corpus
		Overall	Moderate-Severe	Overall	Moderate-Severe	Overall	Duodenal	Overall	Moderate-Severe	PGI/PGII	Overall	Moderate-Severe	Overall
H, K-ATPase antibodies		↑		↑			↑	↑	↑	↓			↑
*H. pylori* infection		↑	↑	↑	↑	↑	↑	↑		↓	↑		
Gender = Male							↓						
BMI						↓	↓				↓		
Age	↑			↑	↑			↑	↑	↓	↑	↑	↑
Smoker							↑					↑	
Alcohol consumption (weekly)			↑										
Blood group O comp. to A	↑												
Blood group AB comp. to A					↑		↑						
DRB1*01								↓					
DRB1*03	↓												
DRB1*04											↓		
DRB1*08											↓		
IL1RN L2 comp. to 22											↑		
IL1B -31 TC comp. to CC			↓										

Results for the ratio PGI/PGII are obtained using the GLM analysis and the remaining results are obtained using the logistic regression model. An up arrow (↑) represents a significant positive correlation, and a down arrow (↓) represents a significant negative correlation for each independent variable. No arrow indicates a lack of significant association. Atrophy and inflammation are graded histologically according to the Sydney system.

#### Relations between the analyzed polymorphisms and H. pylori status

Results from binary logistic regression analysis of *H. pylori* infection (170 positive cases of 442 examined subjects) are presented in Table 2. *H. pylori* infection was positively related to age (odds ratio (OR) 95% CI: 1.027 - 1.066) and negatively related to presence of the HLA-DRB1*03 allele (OR 95% CI: 0.388 - 0.989). There was an overall significant difference between blood groups (P = 0.047), and in comparisons between pairs of blood groups with A as reference category, blood group O showed a higher risk of *H. pylori* infection (OR 95% CI: 1.121 - 2.677).

**Table 2:** Result From Binary Logistic Regression With of *H. Pylori* Infection as Dependent Variable

Dependent variable	No. of positive cases	No. of included cases	Variables in the equation	P-value^#^	95% CI for OR	
					Lower	Upper
*H. pylori* infection	170	442	Age	0.000	1.027	1.066
			ABO	0.047		
			ABO (B)	0.758	0.416	1.894
			ABO (O)	0.013	1.121	2.677
			ABO (AB)	0.160	0.797	3.949
			DRB1*03	0.045	0.388	0.989

Hosmer and Lemeshow goodness of fit test was used as indicator of the validity of the equation at the last step of iterations; ^#^The significance level was set to P < 0.050.

#### Relations between the analyzed polymorphisms and the degree of chronic inflammation of the gastric mucosa

Results from the binary logistic regression analysis of the grade of chronic inflammation in the corpus and antrum mucosa (overall or moderate to severe) are shown in Table 3. Overall inflammation (grade 1-3) in the corpus (181 cases of 442 examined subjects) was associated with elevated levels of H, K-ATPase antibodies (OR 95% CI: 1.017 - 1.061), *H. pylori* infection (OR 95% CI: 44.090 - 170.14), and age (OR 95% CI: 1.007 - 1.072). When including only individuals with histologically determined moderate to severe (grade 2-3) inflammation of the corpus mucosa (42/442 individuals), there was a positive association with *H. pylori* infection (OR 95% CI: 3.028 - 15.713), age (OR 95% CI: 1.013 - 1.088) and a significant difference between ABO blood groups (P = 0.049), where the AB blood group showed higher risk of moderate to severe inflammation of the corpus mucosa than group A (OR 95% CI: 1.400 - 11.705).

**Table 3:** Result From Binary Logistic Regression Analysis With Chronic Inflammation as Dependent Variable

Dependent variable	No. of positive cases	No. of included cases	Variables in the equation	P-value^b^	95% CI for OR	
					Lower	Upper
Inflammation overall grade 1-3 of the corpus	181	442	H, K-ATPase ab	0.001	1.017	1.061
			*H. pylori* inf.	0.000	44.090	170.14
			Age	0.015	1.007	1.072
Inflammation grade 2-3 of the corpus	42	442	*H. pylori* inf.	0.000	3.028	15.713
			Age	0.007	1.013	1.088
			ABO (A)	0.049		
			ABO(B)	0.857	0.291	4.408
			ABO(O)	0.949	0.448	2.123
			ABO(AB)	0.010	1.400	11.705
Inflammation overall (grade 1-3) of the antrum	181	442	H, K-ATPase ab	0.019	1.003	1.038
			*H. pylori* inf.	0.000	241.34	2331.7
Inflammation grade 2-3 of the antrum	114	442	*H. pylori* inf.	0.000	55.397	509.42
			Alcohol cons. (weekly)	0.016	1.247	8.277
			IL1B-31 (CC)	0.026		
			IL1B-31 (TC)	0.011	0.094	0.733
			IL1B-31 (TT)	0.140	0.169	1.285

^a^Hosmer and Lemeshow goodness of fit test was used as indicator of the validity of the equation at the last step of iterations; ^b^The significance level was set to P < 0.050.

Overall inflammation (grade 1-3) in the antrum (181/442 subjects) was associated with elevated levels of H, K-ATPase antibodies (OR 95% CI: 1.003 - 1.038), and *H. pylori* infection (OR 95% CI: 241.34 - 2331.7). Moderate to severe (grade 2-3) inflammation of the antrum (114/442 subjects) was positively related to *H. pylori* infection (OR 95% CI: 55.397 - 509.42) and alcohol consumption on a weekly basis (OR 95% CI: 1.247 - 8.277). There was a significant difference between the IL1β-31 genotypes (P = 0.026) and the TC genotype showed lower risk of moderate to severe inflammation of the antrum than the CC genotype (OR 95% CI: 0.094 - 0.733).

#### Relations between the analyzed polymorphisms and the prevalence of ulcer

Using the binary logistic regression model, peptic ulcer, location disregarded (38/442 subjects), was significantly positively associated with *H. pylori* infection (OR 95% CI: 4.576 - 28.052) and with decreasing BMI (OR 95% CI: 0.725 - 0.938; Table 4). Duodenal ulcer specifically (18/442 subjects) was positively related to the levels of H, K-ATPase antibodies (OR 95% CI: 1.004 - 1.042) and *H. pylori* infection (OR 95% CI: 1.678 - 18.377), and more prevalent in women (OR 95% CI: 0.077 - 0.885), and smokers (OR 95% CI: 1.246 - 11.636). There was a significant difference between ABO blood groups (P = 0.014), and group AB showed higher risk for duodenal ulcer than blood group A (OR 95% CI: 2.284 - 46.953). There was also a negative relation between BMI (OR 95% CI: 0.663 - 0.950) and the occurrence of duodenal ulcer.

**Table 4:** Result From Binary Logistic Regression for Peptic Ulcer

Dependent variable	No. of positive cases	No. of included cases^*^	Variables in the equation	P-value^b^	95% CI for OR	
					Lower	Upper
Ulcer overall	38	442	*H. pylori* inf.	0.000	4.576	28.052
			BMI	0.003	0.725	0.938
			H, K-ATPase ab	0.013	1.005	1.042
Duodenal ulcer	18	442	*H. pylori* inf.	0.005	1.678	18.377
			Gender	0.031	0.077	0.885
			BMI	0.012	0.663	0.950
			Smoker	0.019	1.246	11.636
			ABO (A)	0.014		
			ABO (B)	0.826	0.061	9.362
			ABO (O)	0.416	0.477	6.017
			ABO (AB)	0.002	2.284	46.953

^*^In some cases data were not available for all variables; ^a^Hosmer and Lemeshow goodness of fit test was used as indicator of the validity of the equation at the last step of iterations; ^b^The significance level was set to P < 0.050.

#### Relations between the analyzed polymorphisms and the prevalence of atrophy in the corpus mucosa

Results from the binary logistic regression analysis of atrophy of the corpus mucosa (overall or moderate to severe) are shown in Table 5. Results from the statistical analysis using the serum level of the ratio PGI/PGII as surrogate marker for atrophy of the corpus (as determined histomorphologically) are presented in Table 6. Atrophy of the corpus mucosa (41/442 subjects) was positively related to the levels of H, K-ATPase antibodies (OR 95% CI: 1.016 - 1.042), *H. pylori* infection (OR 95% CI: 1.772 - 8.520) and age (OR 95% CI: 1.018 - 1.101), and negatively related to the presence of HLA-DRB1*01 (OR 95% CI: 0.050 - 0.837). When including only individuals with histologically determined moderate to severe atrophy of the corpus (17/442 subjects), there was a significantly positive association with the H, K-ATPase antibody levels (OR 95% CI: 1.010 - 1.038) and age (OR 95% CI: 1.099 - 1.323).

**Table 5:** Result From Binary Logistic Regression Analysis With Atrophy of the Corpus Mucosa as Dependent Variable

Dependent variable	No. of positive cases	No. of included cases	Variables in the equation	P-value^b^	95% CI for OR	
					Lower	Upper
Atrophy overall (grade 1-3) of the corpus^c^	41	442	H, K-ATPase ab	0.000	1.016	1.042
			*H. pylori* inf.	0.001	1.772	8.520
			Age	0.004	1.018	1.101
			DRB1*01	0.027	0.050	0.837
Moderate to severe atrophy of the corpus^d^	17	442	H, K-ATPase ab	0.001	1.010	1.038
			Age	0.000	1.099	1.323

**Table 6:** Result From GLM Analysis Using the Ratio Pepsinogen I/Pepsinogen II (PGI/PGII) as Surrogate Marker for Atrophy of the Corpus Mucosa

Dependent variable	Variables in the equation	Coef.	SE Coef.	T	P-value^a^	95% CI	
						Lower	Upper
PGI/PGII	H, K-ATPase ab	-0.047523	0.008793	-5.40	0.000	-0.06480	-0.03024
	*H. pylori* inf.	-3.5956	0.3385	-10.62	0.000	-4.26080	-2.93031
	Age	-0.03434	0.01545	-2.22	0.027	-0.06470	-0.00397

^a^The significance level was set to P < 0.050.

Using the ratio PGI/PGII as surrogate marker of histologically classified atrophy, GLM analysis revealed a significant negative association between the titer of H, K-ATPase antibodies (95% CI: -0.06480 - -0.03024), *H. pylori* infection (95% CI: -4.26080 - -2.93031), and age (95% CI: -0.06470 - -0.00397), and the ratio PGI/PGII.

#### Relations between the analyzed polymorphisms and the presence of IM in the corpus and antrum mucosa

IM in the corpus (22 cases with grade 1-3 out of 442 subjects) was positively associated with the H, K-ATPase antibody titer (OR 95% CI: 1.018 - 1.042) and age (OR 95% CI: 1.070 - 1.220). Only 5 cases of moderate to severe IM in the corpus mucosa were found in this population. The Hosmer and Lemenshow goodness of fit test was significant, indicating a non-valid equation, and the results were excluded from further analysis.

IM in the antrum (89 cases with grade 1-3 out of 442 subjects) was positively associated with *H. pylori* infection (OR 95% CI: 9.675 - 38.480), and decreasing BMI (OR 95% CI: 0.758 - 0.927), increasing age (OR 95% CI: 1.003 - 1.061), and negatively related to carriage of the HLA DRB1 alleles *04 (OR 95% CI: 0.234-0.831) and *08 (OR 95% CI: 0.013 - 0.915) (Table 7). There was an overall significant difference between IL1RN genotypes (P = 0.013), and IL1RN*L2 carriers showed a higher risk of IM in the antrum than genotype *22 carriers (OR 95% CI: 1.570 - 15.878).

**Table 7:** Result From Binary Logistic Regression Analysis With Intestinal Metaplasia in the Corpus or Antrum Mucosa as Dependent Variable

Dependent variable	No. of positive cases	No. of included cases	Variables in the equation	P-value^b^	95% CI for OR	
					Lower	Upper
IM in corpus, grade 1-3	22	442	H, K-ATPase ab	0.001	1.018	1.042
			Age	0.000	1.070	1.220
IM in corpus, grade 2-3	5	442	Excluded due to sign HL test			
IM in antrum, grade 1-3	89	442	*H. pylor*i inf.	0.000	9.675	38.480
			BMI	0.001	0.758	0.927
			Age	0.031	1.003	1.061
			DRB1*04	0.011	0.234	0.831
			DRB1*08	0.041	0.013	0.915
			IL1RN (22)	0.013		
			IL1RN (L2)	0.006	1.570	15.878
			IL1RN (LL)	0.084	0.876	8.172
IM in antrum, grade 2-3	11	442	Age	0.007	1.027	1.188
			Smoking	0.030	1.155	17.446

^a^Hosmer and Lemeshow goodness of fit test was used as indicator of the validity of the equation at the last step of iterations; ^b^The significance level was set to P < 0.050.

Considering moderate to severe IM in the antrum (grade 2-3; 11 cases), there was a significant relation to increasing age (OR 95% CI: 1.027 - 1.188) and smoking (OR 95% CI: 1.155 - 17.446) (Table 7). None of the genotypes was associated with moderate to severe IM.

## Discussion

The aim of this study was to explore possible associations between IL-1B-31T/C, IL1RN VNTR alleles, IFNGR1-56C/T, the HLA DRB1 alleles, blood group and life style factors and *H. pylori* infection, the occurrence of peptic ulcer, grade of gastric mucosal inflammation, atrophy, and IM in a prospective population based cohort in Sweden. The findings are summarized in Table 1. As stated in our first publication concerning this population [17], there was a modest overrepresentation of digestive symptoms among those volunteers that agreed to participate in the screening study compared to an age and sex matched population, which was not being asked to participate in any examinations.

Several human cytokine genes have been thoroughly studied with respect to their influence on the host-microbial interaction of the *H. pylori* colonization, establishment of infection and the long-term outcome such as duodenal ulcer and the premalignant condition atrophic gastritis. The findings in these studies are not unequivocal and differ in various populations [7]. Two well-studied genes are the IL1β and its receptor antagonist IL1RN. El-Omar et al (2000) [4] showed that mutations in the IL1β gene promoter position -31 (C instead of T) and the short form of the *IL1RN* VNTR (allele No. 2) were significantly associated with increased risk of developing gastric carcinoma following *H. pylori* infection. In the present study, using logistic regression analysis, no significant association was found between the *IL1B*-31T/C and *H. pylori* infection, peptic ulcer, gastric atrophy, or IM (Table 1). However, a negative correlation between the *IL1B*-31TC genotype was found for moderate to severe inflammation in the antral mucosa, but not for overall inflammation (grade 1-3 according to the Sydney system) or for any degree of inflammation in the corpus mucosa.

No associations were found between IL1RN VNTR alleles and *H. pylori* infection, any grade of inflammation, ulcer, or atrophy. However, in pairwise comparison between IL1RN genotypes, the *L2 genotype showed a higher risk for IM (grade 1-3) in the antrum (Table 7) than *22. No relation to the IL1RN VNTR alleles was seen when including only cases with moderate to severe IM (11 cases). When searching for papers reporting studies of IL1RN and IM, we found two studies reporting no association between IL1RN*2 genotype and IM [22, 23], and one study reporting an association between IL1RN*2 and IM in an Italian population [24]. However, they do not report any possible association between the IL1RN*L2 genotype and IM, and our findings cannot be verified.

Discussion of the results from each step is presented below in the following order: 1) *H. pylori* infection; 2) grade of inflammation; 3) ulcer; 4) atrophy of the corpus mucosa; and 5) IM of the corpus and antrum mucosa.

Beside a positive relation to age, we found a higher risk of *H. pylori* infection for the ABO blood group O (compared to group A). We also noted a negative relation between the DRB1*03 allele and *H. pylori* infection. This negative relation was also found by Veneri et al (2005) [25], but their study included only 52 patients with idiopathic thrombocytopenic purpura (of whom 34 had *H. pylori* infection). Kunstmann et al (2002) [26] found no such relation in a German cohort. The positive association between blood group O and *H. pylori* infection and gastroduodenal diseases has been described and characterized in other studies [2, 27, 28], but discrepant data regarding this are on record [29, 30].

Overall inflammation (grade 1-3) of the corpus mucosa was related to increased titer of H, K-ATPase antibodies, *H. pylori* infection and increasing age. If only moderate to severe (grade 2-3) inflammation of the corpus was included in the statistical model, no relation to the titer of anti-H, K-ATPase antibodies was found, but there was, however, a higher risk of inflammation of the corpus for blood group AB than group A. Previously, blood group AB has been found to be negatively associated with *H. pylori* infection [27], a relation that was not observed in this study. To the best of our knowledge, this is the first time a positive relation between moderate to severe inflammation of the corpus mucosa and blood group AB has been demonstrated.

We found a positive relation between overall inflammation (grade 1-3) in the antrum and *H. pylori* infection and increasing titer of H, K-ATPase antibodies. However, there was no association between the presence of moderate to severe (grade 2-3) antral inflammation and the titer of anti-H, K-ATPase antibodies. Instead, we noted a relation between weekly alcohol consumption and lower risk of inflammation for the IL1B-31 TC genotype (compared to the CC genotype) (Table 3). To our knowledge, this relation has not been described previously.

For peptic ulcer, location disregarded, a positive relation was found between *H. pylori* infection and low BMI. Regarding individuals with ulcers exclusively located to the duodenum, we found associations to H, K-ATPase antibodies, *H. pylori* infection, smoking, blood group AB (compared to group A), female gender and decreasing BMI (Table 1). Sierra et al (2008) [31] also found an association between duodenal ulcer and smoking, but no association between IL1-RN and duodenal ulcer in a Costa Rican dyspeptic population. This is in agreement with our data. The relation to blood group AB is discordant with the findings of previous investigators who have demonstrated an association between blood group O and the prevalence of peptic ulcer [32] (reviewed by Anstee and Clarke et al [28, 33]). However, others found no association between ABO blood groups and ulcer [29].

Regarding atrophy of the corpus mucosa (grade 1-3 according to the Sydney system), a positive relation was found between H, K-ATPase antibodies, *H. pylori* infection and increasing age, and a negative relation to DRB1*01 carriage. A negative relation between the DRB1*01 allele and atrophic gastritis has also been demonstrated by Lahner et al (2010) [34] with an OR of 0.27 (95% CI 0.08-0.089; P = 0.02). However, we did not find any association between the DRB1*03 or *04 and atrophic gastritis as found by Lahner et al (2010) [34]. When including only moderate to severe cases of atrophy (grade 2-3) of the corpus mucosa, we found associations to atrophy and H, K-ATPase antibodies and increasing age. The PGI/PGII ratio is commonly used as a screening marker for AG [35], where a low-value ratio indicates presence of AG. When using the ratio PGI/PGII as surrogate marker for AG of the corpus, a negative association between H, K-ATPase antibody titer, *H. pylori* infection, and age and increasing ratio (of PGI/PGII) was noted. This is in accordance with our results for AG overall (grade 1-3 according to the Sydney system), except that no association between PGI/PGII ratio and DRB1*01 allele carriage emerged.

In this study, IM in the corpus mucosa of grade 1-3 (according to the Sydney system) was found in 22 cases and this was related to increasing titer of H, K-ATPase antibodies and increasing age (Table 7). Only 5 cases with moderate to severe IM were found in the corpus mucosa of this population. In the statistical analysis, the Hosmer and Lemenshow goodness of fit test was significant, indicating a non-valid equation, and the results were excluded from further analysis.

IM overall (grade 1-3) in the antrum mucosa was found in 89 subjects and was associated with *H. pylori* infection, decreasing BMI, increasing age, the HLA-DRB1 alleles *04 and *08 and the IL1RN *22 (the latter discussed above). IM of moderate to severe grade (2-3) in the antrum mucosa (11 cases) was only associated with increasing age and smoking. The association between IM and increasing age and smoking is well established [36]. The relation of IM in the antrum mucosa and the two HLA-DRB1 alleles *04 and *08 has to our knowledge not been reported before. The findings could not be confirmed for cases with moderate to severe IM in the antrum mucosa (Table 7).

Concerning IFNGR1-56C/T, we found no significant relation to any of the diseases studied here.

In summary, in this prospective Swedish general population based cohort study, we found positive associations between the presence of blood group O and *H. pylori* infection. We also found a positive association between blood group AB and moderate to severe inflammation of the corpus mucosa, as well as the occurrence of duodenal ulcer. For the HLA DRB1 alleles, a negative association was found between DRB1*03 and *H. pylori* infection, and for DRB1*04 and *08 in relation to IM (grade 1-3) of the antrum mucosa. A higher risk of overall IM in the antrum mucosa for heterozygous IL1RN*L2 carriers than *22 carriers, and a lower risk of moderate-severe inflammation of the antrum for IL1β-31 TC carriers than CC carriers, were established.

No other significant correlations were identified between the occurrence of the polymorphisms together with life style factors on the one hand, and the occurrence of *H. pylori* infection, peptic ulcer, and the grade of inflammation, atrophy and IM of the gastric mucosa, on the other hand.

*H. pylori* infection and age were clearly associated to most of the gastrointestinal diseases studied here. The IL1RN VNTR and the IL1β-31 alleles seem to be associated with IM of the corpus mucosa and the grade of inflammation of the antrum, respectively. However, no unambiguous correlations could be identified between the host polymorphisms and the occurrence of *H. pylori* infection, peptic ulcer, and the grade of inflammation, atrophy and IM of the gastric mucosa.
